# Anatomy, Physiology and Histology

**Published:** 1875-12

**Authors:** 


### V. Anatomy, Physiology and Histology.

1. *Function of the Levator Ani Muscle.* D. Budge. (*Berliner Klin. Wochenschrift*, No. 27, 1875.)

This muscle is believed by some to raise and dilate the anus, by others to be a second sphincter. Its relation to the pelvic fascia must be studied in order to arrive at a correct opinion about its function. Its fibres spring from the tendinous arch of the pelvic fascia, at the horizontal ramus of the pubis, the ischial spine, and the anterior end of the coccyx. The fibres while spreading over the movable portion of the pelvic fascia, surround the lower part of the rectum in such a way that this is constricted by the contracting muscle. And B. has proved by experiments on animals recently killed, that the excitation of this muscle, produces a perfect obstruction to the passage of water through the rectum. It is the levator muscle, and not the superior sphincter ani of Nélaton, which effects a retention of fæces in case of destruction of the sphincter ani.

2. *Narcotism by the Products of Tissue Change after Fatigue.* PREYER. (*Times and Gazette*, August 28.)

The fact that severe fatigue, either of the muscular or nervous system, predisposes to sleep, has led Dr. W. Preyer, of Jena, to make experiments with those substances which are most likely to be formed in the tissues after such exertion, especially lactic acid, to see whether they possess any narcotic properties. He finds, after a long series of observations and experiments, that lactate of soda, either subcutaneously injected in a concentrated aqueous solution, or introduced into the empty stomach in large doses, frequently induces sleep, if the subject is kept quiet and undisturbed. There are, however, considerable individual differences in its effects, both on animals and men, not only as to the time of onset of sleep, but also as to its duration and intensity. Young and small animals are affected more easily than old and large animals. Preyer finds that the exhibition of such liquids as are favorable to an abundant production of lactate of soda in the intestinal canal—*e. g.*, concentrated syrup or copious draughts of sour milk, and of whey—in many cases give rise to drowsiness, and even to actual sleep ; and he thinks this fact might be of practical application in some diseases, where morphia and chloral are now exclusively ordered. Lactate of soda in doses much larger than are required to induce sleep, increases the number of respirations and also their depth ; and reflex irritability, and (in warm-blooded animals) the temperature, are diminished. Dr. Preyer, whose communication will be found in *Centralblatt*, (No. 35, August 7), from his experiments on animals is strongly opposed to the lactates of potash, magnesia and lime for narcotic purposes, in the human subject.

3. *New Test for Amyloid Tissues.* PROF. HESCHL. (*Wiener Med. Wochenschrift*, No. 32, 1875.)

In 1871, the Professor discovered in his writing fluid, a very delicate test for amyloid degeneration of tissues.

This ink, known as Leonhard's (Dresden) violet ink, is a compound of aniline blue and aniline red, carefully prepared, and mixed with such substances as make this compound good for a writing fluid. Mixed with a little glycerine it stains amyloid substance a beautiful red color, while all other tissues exhibit a blue tint. And this reaction is so sensitive that it will show by a red color, the slightest degree of amyloid degeneration, even where the test with iodine and sulphuric acid fails. The slices prepared for the microscope are covered with a drop of the fluid; the glass is superimposed and by means of distilled water, the superfluous coloring matter is washed out from under the glass. If by too long an exposure to the ink, the normal tissues have become too opaque, the color can be extracted by water. Such preparations may be preserved in every liquid used for this purpose. Another advantage of this new test, is that it can be employed also without failure in substances preserved in alcohol, or Müller's solution.

4. *A New Process for Staining Tissues.* F. E. Hoggan, M.D. (*Medical News; Brit. Med. Jour.*, August 28, 1875.)

Mrs. Frances Elizabeth Hoggan, M.D., London, discussed her process for staining tissues at the late meeting of the British Medical Association. The process recommended itself principally on account of the property it possessed of staining the substance of the cell, as well as the nucleus and nucleolus, and because it gave the best results where carminate of ammonia failed. It consisted in first pouring over the specimen (after treating it with water and with methylated spirit) a one per cent. solution of perchloride of iron; and, in a few minutes afterwards, a few drops of a two per cent. solution of pyrogallic acid, both solutions being made in distilled water. A practical demonstration of the process was given by Mrs. Hoggan.

5. *Changes Produced by the Circulation in the Volume of Members.*

At the late "French Association for the Advancement of Science," M. Franck made a very interesting communication upon the above subject. It is thus epitomized by the *Progrès Médical*,* Sept. 4, 1875 :

"If the hand be plunged into a vessel of water, and care be taken to exclude the air, the level of the liquid will be seen to rise with each augmentation of volume of the immersed member. If a vertical tube be adapted to this vessel the changes in the level will be more appreciable and can be recorded in the form of a tracing. It is possible, at the same time that the changes of volume of one arm for example are being studied, to take, upon the same person, a tracing of the pulse of the other arm, a tracing of the respiration and one of the beatings of the heart.

"Now, it can be seen that the tracing of the augmentation of volume and that of the pulse give a parallel curve; but the trace of augmentation of volume is modified by the respiration. The curve of the respiration does not exactly correspond with the deviations that the respiration causes the curve of the changes in the volume to undergo. There is not perfect synchronism, since the two lines cross each other. Under the influence of effort, the outline of the tracing of the augmentation of volume rises rapidly, then it remains stationary whilst the amplitude of the pulsations diminishes. If, by a ligature moderately tightened, the return of venous blood is prevented, the tracing takes exactly the form of a pair of stairs, the steps of which progressively diminish in height. It becomes a horizontal line when the pressure in the veins becomes equal to the cardiac impulse.

"If the two femoral arteries be compressed, the tracing rises during two beats. During the first beat, there is a movement of quick ascension, corresponding to a brusque suppression of a large vascular area. During the second beat, there is a progressively ascending movement, corresponding to the return of venous blood from the lower limbs, a period during which the inferior members become empty of venous blood and do not receive any (blood). If the arm, opposite to the one experimented with, be elevated, the tracing rises, the blood contained in the uplifted arm falling back of its own weight and augmenting the pressure in the rest of the circulatory system.

"When the brachial artery of the arm experimented with is compressed, the tracing descends, and returns again when compression ceases, and exceeds the former level. The volume of the hand is perhaps influenced by vaso motor action; if the hand be cooled, a diminution of the volume ensues. It is precisely the same—remarkable fact!—if the opposite hand be cooled. The explanation of this fact is by no means clear. It is supposed that it occurs from reflex action."

